# South American Values of the Optical Straylight Function

**DOI:** 10.3390/vision4010002

**Published:** 2019-12-24

**Authors:** Emilia Longhi Bitencourt, Nestor Norio Oiwa, Dora Fix Ventura, Marcelo Fernandes Costa

**Affiliations:** 1 Departamento de Psicologia Experimental, Instituto de Psicologia, Universidade de São Paulo, São Paulo 05508030, Brazil; longhi@gmail.com (E.L.B.); nnorio@usp.br (N.N.O.); dventura@usp.br (D.F.V.); 2 Núcleo de Neurociências e Comportamento e Neurociências Aplicada, Universidade de São Paulo, São Paulo 05508030, Brazil

**Keywords:** retinal straylight, C-Quant, psychophysics, ocular lens, vision aging

## Abstract

Purpose: To access retinal straylight in a Brazilian sample and to compare it with European norms. Methods: Absolute Straylight was assessed using C-Quant that uses an adaptive staircase based on a 2-Alternated Forced Choice task. A young (22.2 ± 2.4 yrs, *n* = 20) and an old group (53.8 ± 7.4 yrs, *n* = 21) of subjects were tested. All refractive errors were corrected in the C-Quant device, and no subjects had ocular diseases or vision-threatening conditions (e.g., diabetes, unregulated blood pressure, high intraocular pressure, visible cataract). Eighty-five percent of all subjects in each age group had dark-pigmented eyes. Each eye was tested 3 times, yielding 6 straylight values (s). Only data fulfilling C-Quant reliability criteria were included. Results: There were no statistical differences between the three attempts on each eye (ANOVA, *F* = 0.993, *p* > 0.936) and between the two groups (ANOVA, *F* = 0.893, *p* > 0.725). Straylight values (s) were fit with an empirical equation to compare to European norms. There were no statistical differences between Brazilian straylight values and European norms for either young or old age groups (ANOVA, *F* = 5.114, *p* > 0.993). However, there was a tendency for our s values to be higher than the European norms, consistent with young Brazilian eyes having more light-scattering than age-matched European eyes. Conclusions: Consistent with European norms, light-scattering increases with age in the Brazilian sample. This increase is thought to be due, in large part, to age-related changes in lens structure and density. Although the differences between the populations are not significant, the tendency for Brazilian data to have higher *s* values than European values, especially at young subjects, is in the opposite direction from that expected from a dark-eyed population. This suggests the hypothesis that latitude-dependent (Sao Paulo, latitude 23° S, European latitudes between 40° N to 55° N) differences in the light environment could be associated with differences in s values.

## 1. Introduction

Among several visual functions, retinal straylight has a crucial part in everyday life, because of its role in driving, mainly at night. The CIE (*Comission International d’Eclairage*) defines disability glare as retinal straylight, a result of studies that showed that disability glare can be understood on the basis of light scattering in the eye [1]. Light scattering happens in all eyes because the scattering is caused by light interference with biological particles (protein, cells) present in the eye, changing its direction. The amount of retinal straylight in the young, healthy human eye is due mainly to three different causes: 1/3 is caused by the cornea, 1/3 by the lens, and 1/3 by the iris, sclera, and fundus [2]. Because of that, any change in all these parts may increase retinal straylight, and individual differences, such as iris pigmentation and amount of pigment at the fundus, can change the normal amount of retinal straylight; for instance, without so many pigments at the iris, light-colored eyes may have higher values of straylight when comparing to dark-colored eyes [3]. 

Many conditions can increase retinal straylight, such as in vitreous turbidity, corneal disturbances, use of contact lenses and refractive surgery, but the most common condition is the cataract. Because of that, a natural increase of straylight is expected during aging [3]. 

The increase of straylight causes difficulties to see, especially when there is a light source near what is seen [4]. It is a relevant condition during driving, since the headlights of oncoming cars can work as a glare source, leading the driver to experience difficulties seeing any objects (including people) that might be in front of the car, and it is harder during the night, when the headlights may be one of the only light sources. This condition is not exclusive for driving, and its effects can be perceived in everyday life, but driving is a critical situation that may cause damage to the driver and others. 

As safety is an important issue in driving, the European Community developed a project between 2003 and 2004 (GLARE Project) [4], testing retinal straylight and other visual functions on 2422 drivers in five European cities (Salzburg, Antwerp, Tübingen, Barcelona, and Amsterdam). Retinal straylight was accessed using a new psychophysical approach, developed by Thomas van den Berg’s group, and this method, called Compensation Comparison [5], was used to create a commercial device to measure retinal straylight, called C-Quant (Oculus Optikgeräte, Germany). As a result of the project, a function was fit to determinate the increase of retinal straylight with age in the general population. The resulting curve is presented in the C-Quant software as expected values of straylight according to age, from 20 to 90 years old, based on a predominant Caucasian, light-pigmented eye sample.

In order to determine data reliability using C-Quant, two criteria were built inside the software, based on the psychophysical curve obtained with the GLARE Project–Esd and Q. Those criteria were tested to confirm the reliability of C-Quant results [6,7].

Several studies showed the independence between retinal straylight and other visual functions, as visual acuity and contrast sensitivity [8]; there was no relation also with slit-lamp based examinations [8,9] and with normal pupil diameter at photopic conditions [10,11].

The purpose of this study was to access retinal straylight in a Brazilian sample, and, by comparing the results with the European norms, to observe possible straylight differences between the two populations that might be due to pigment characteristics of the Brazilian sample. 

## 2. Methods

**Figure 1:**
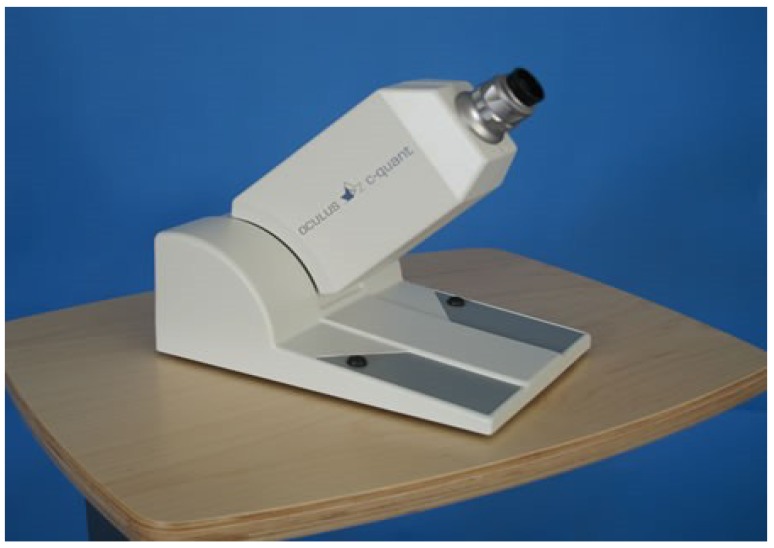
C-Quant equipment. Left and right black buttons located on the silver area at the base were used to the 2-Alternated Forced Choice left–right judgment.

Absolute retinal straylight was assessed using C-Quant (Oculus Optikgeräte, Germany—Figure 1) that uses a 2-alternative forced-choice (2AFC) adaptive staircase called Compensation Comparison Method [5]. The visual field is 14° of visual angle. The illumination was produced by an LED light with a maximum luminance of 300 cd m^−2^. For more comfort of the participant, the device inclination could be angled between 35° to 55°. 

**Figure 2:**
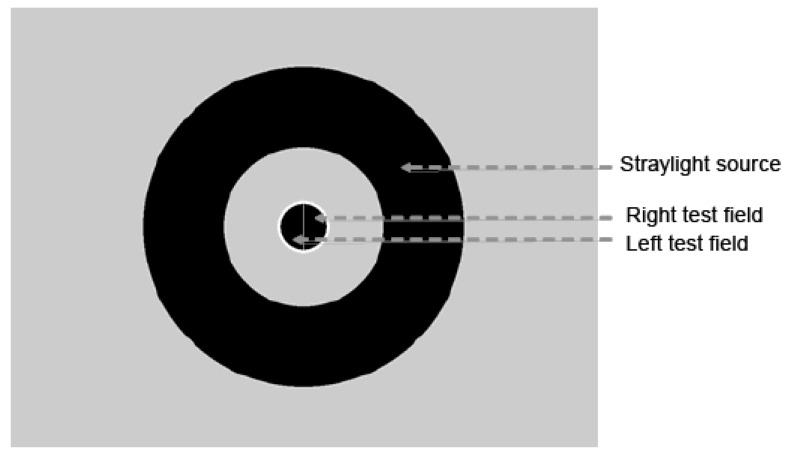
An example of test fields observed inside the C-Quant ocular opening. During the experiment, the outside circle is illuminated to generate the retinal straylight as the participant judging in which the central circle hemifield the light flickers stronger.

The task for the subject is to choose, from the test field, the side of the central circle that flickers more strongly, and the response is giving by pressing buttons in the device (one on each side). To determinate, the straylight value, a straylight source of light is presented in the surround of the visual field, and in the counter phase, there is a compensation light presented in one side of the central circle (test field). The outside circle is illuminated flickering to induce flickering straylight in the center. In one half-field counter-phase flicker is added. The participant judged which side flickers stronger. Both straylight and compensations lights change intensity during the test. As a result, a psychometric function is built according to the subject’s responses, and the straylight value is the minimum point of this curve (Figure 2).

The visual function was measured on 41 subjects, divided into group 1 (18–30 yrs; mean 22.2 ± 2.4 yrs, *n* = 20) and group 2 (40–60 yrs; mean 53.8 ± 7.4 yrs, *n* = 21). All subjects lived in São Paulo, Brazil, and they had no ocular diseases or vision-threatening conditions (e.g., diabetes, unregulated blood pressure, high intraocular pressure, visible cataract) and most of them (85%) had dark-pigmented eyes. When necessary, refractive errors were corrected in the C-Quant apparatus, and the subjects that wore contact lenses were asked to stop using them for at least 7 days before the test and in the day of the experiment to not use any product in the face as makeup, solutions, creams, and cosmetic oils. The inclusion criteria were 20/20 or better visual acuity and no more than 1.0 spherical diopters of refractive error, since according to Rozema et al. [12], there is an early increase in the straylight value. This study was approved by the Institute of Psychology Ethical Committee (Prot. # 2507–CEPH-IP 13/11/2007), and a Consent Term was presented to all the subjects and signed before the test. 

Each eye was tested 3 times, yielding 6 straylight values (s) per subject. Only data fulfilling C-Quant reliability criteria (Esd < 0.08, *Q* > 1.0) were included. Testing was performed monocularly at environmental light conditions, alternating right and left eyes, and the first eye tested was chosen by the subject.

For sample control and characterization, pupil diameter was measured, at environmental light condition, before the test. Also, a questionnaire was answered by all subjects, concerning health conditions and everyday habits, such as smoking and drinking habits, since those factors might influence visual conditions.

For data analysis, the program Statistica (Statistica 8.0, Statsoft, Tulsa, OK, USA) was used. A full descriptive analysis was performed. Comparisons between the groups were done by One-Way ANOVA. For the Principal Components Analysis the eigenvalues indicating the importance of the respective factors (eye tested) in explaining the variation of the data.

## 3. Results

Retinal straylight was measured six times, as described, but some subjects could not respond properly, according to C-Quant reliablity criteria, in all attempts. It might be because the trial can cause some eye fatigue, which can make the response more difficult for older people, for instance, who have usually higher straylight values caused by aging. So, in group 1 (18–30 yrs) all subjects perform the test six times, three times each eye, but in group 2 (40–60 yrs) not all subjects could do it six times. In some cases, more attempts were made to try to reach the six valid results, but in other cases, the performance decreased so badly that was impossible to continue the trial. Since only results that fulfilled the reliability criteria were used, in group 2, not all the subjects had six valid results.

**Figure 3:**
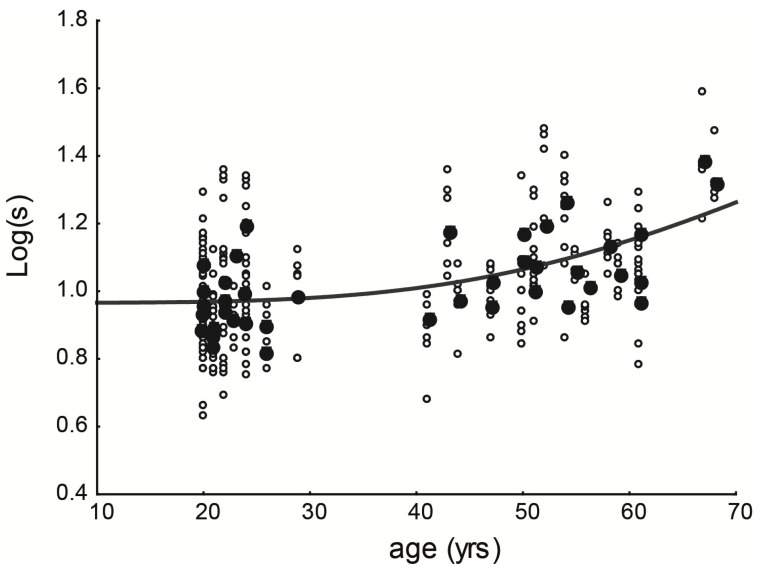
Straylight values from both groups. Dark-grey line indicates the function adjusted to the data: Log(*s*) = log(9.25 × (1 + (age/70.35)^4^)).

Figure 3 shows all data from the two groups, with all valid attempts. It is possible to see that the data are spread in both groups, showing very similar values. The function that fits the data was calculated, and it is represented by the red line. The function is Log(*s*) = log(9.25 × (1 + (age/70.35)^4^)).

No statistical differences were found between the three attempts in each eye (ANOVA, *F* = 0.993, *p* > 0.936) neither comparing both eyes at the same subject (ANOVA, *F* = 0.893, *p* > 0.725). Apparently, although the data seems scattered, statistically all attempts from the same subject, in both eyes, are equivalent.

**Figure 4:**
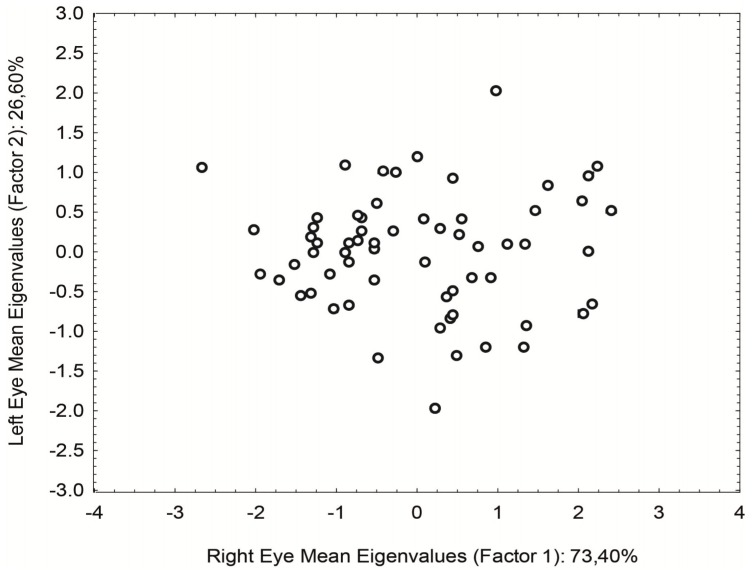
Dispersion of the straylight values from the mean of the 3 measurements performed in right (Factor 1) and left eyes (Factor 2) resulting from the Principal Components Analysis. The right eye showed a higher contribution to the straylight measured.

In an attempt to decrease the data scattering, a principal component analysis (PCA) in a covariance matrix was used to combine measures from both eyes into one single value. That transformed the up-to-six results for each subject into one value, considering the contribution of both eyes. The contribution of the right eyes seemed to be bigger than the left eye in the sample (Eigenvalues: right eye = 0.833; left eye = 0.554) Figure 4.

PCA also gives us the difference between eyes. In this case, the eigenvalues for the right eye = −0.554 and left eye = 0.833. We find a statistical difference concerning the data dispersion with *F* = 2.7 and *p* = 0.016 between groups 1 and 2. The straylight of both eyes became more similar to age. 

**Figure 5:**
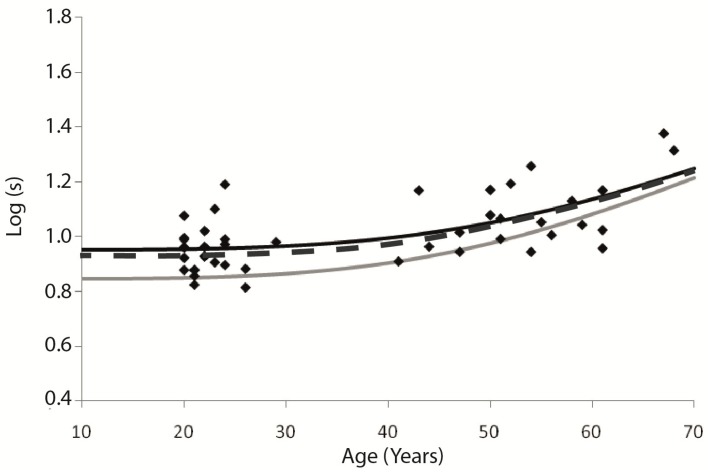
Straylight values after combining the same subject’s measurements at one point. Each point indicates straylight value combining the left and right eyes from one subject. The dark line indicates the function adjusted to these data. The grey line corresponds to the European norms presented in C-Quant. The dark-grey dashed line indicates the new European data obtained by van den Berg in 2013, which corrected the straylight value closer to our model.

After transforming all subject’s data into one value, it was possible to calculate again the function that fits the new data. Figure 5 shows the new data scattering and the new function (dark line-Log(*s*) = log(8.92 × (1 + (age/70.34)^4^)). It also shows the function representing control data from the device, obtained by a population study (GLARE Project [4]) with 2422 subjects, that took place in Europe during 2002–2004, in which drivers were tested at Salzburg, Antwerp, Tübingen, Barcelona, and Amsterdam. That function, that is used in the device (C-Quant) as normative values according to age, is shown as a grey line, and it can be written as Log(*s*) = log(7 × (1 + (age/65)^4^)) [13].

Although the two functions are different, the data scattering makes it impossible to find statistical differences between the two samples (Brazilian and European), even with the new data sample after PCA analysis (ANOVA, *F* = 5.114, *p* > 0.993). But, besides that, it is possible to see a tendency on Brazilian data to have higher straylight values between younger people, comparing to European sample. It is important to notice that even in the European sample the data scattering was present, as shown by van den Berg and his team [4]. 

## 4. Discussion

Before the tests, it was decided that we would perform the test three times each eye, alternating eyes. In the literature, it is shown that the data acquired by C-Quant are quite reliable [6,7] and one measure each eye should be enough to access retinal straylight. But, during a pilot test performed before the actual data mining, we noticed that the resulting variability could indicate that the subject might have problems responding, and those problems could happen for several reasons, including not seeing the test field because of glare and not understanding the instructions. C-Quant uses a psychophysical methodology that depends entirely on the subject’s response, in which misunderstanding the instructions could result in straylight values that fall far outside the expected value and the procedure should be repeated. 

In order to verify if the instructions had been understood, we chose to perform the task three times, so it was possible for us to see if there was internal coherence between the attempts, even if the straylight value obtained was not expected by age. It could indicate to us that the test instructions had been understood, and the result could be faithful to the eye condition. That seemed especially important when testing older people, who could have no intimacy with the task (pressing buttons). It is very important to alternate eyes because testing the same eye repeatedly without breaks can decrease the performance impressively; although there is no learning effect on the test, there is a fatigue effect caused by several repetitions, and it was observed during our pilot test. As we could tell, a 2-min break seems enough to avoid the fatigue effect to take place.

Comparing the Brazilian and European retinal straylight functions, it is possible to see that they are very similar, but with a different behavior at young ages (before 30); apparently, there is a tendency of higher straylight values at the Brazilian sample. It was very surprising because of the pigment characteristics of the Brazilian sample since it was expected that a dark-eyed population would have lower straylight values. In fact, the pigment characteristics of the European subjects that took part of the GLARE project (Caucasian, light-eye drivers) gave us the first idea of testing a Brazilian sample, because our population is not predominantly Caucasian and light-eyed, and the role of the pigmentation is well known at straylight. We believed that our sample would have lower straylight values, because of that. And, apparently (since there was no statistical significance) it was not confirmed, even with a small sample as ours. More recent data obtained for the light-eyed population found straylight values more similar than our data, which closer to log(s) = 0.9 [14]. Of course, it is not possible to compare a 41-people sample to a population study, but the behavior of the data was very similar, even observing the data scattering. Considering those new data, the results we found are even more similar to those of European norms showing a high consistent methodology used by the C-Quant. 

In order to try to understand the straylight results in our sample, we believe that the higher straylight values presented at group 1, when comparing to European norms, might be due to environment light differences related to latitude. Our research took place in Sao Paulo, at 23° S, and the GLARE Project involved cities between 40° N and 55° N. The amount of UV radiation is different on those latitudes, and closer to equator latitudes have more UV radiation at the environment illumination. It was interesting that this effect only happened in younger subjects, leading us to the hypothesis that, at younger ages (until 30 years old), environmental aspects might rule the straylight, but as people get older, physiological aspects, such as lens aging, become more important. It would explain also why the functions seem to approach one to another after 60 years old.

Our hypothesis needs, for confirmation, to measure retinal straylight in different latitudes, and the amount of UV radiation in each location must be taken into account. Also, it would be interesting to develop a population study in Sao Paulo, in order to compare the two populations (Brazil and Europe) with a similar sample number and perhaps find statistical differences between the two samples. It is important to note that other factors that affects the eye pigmentation like race should also be considered in future studies. 

## References

[1] Vos J.J. (1984). Disability glare—A state of the art report. CIE J..

[2] Coppens J.E., Franssen L., van den Berg T.J.T.P. (2006). Wavelength dependence of intraocular straylight. Exp. Eye Res..

[3] IJspeert J.K., de Waard P.W., van den Berg T.J.T.P., de Jong P.T. (1990). The intraocular straylight function in 129 healthy volunteers; dependence on angle, age and pigmentation. Vis. Res..

[4] Van den Berg T.J.T.P., van Rijn L.J., De Wit G. (2005). Relevance of Glare Sensitivity and Impairment of Visual Function Among European Drivers. http://www.glare.be/publications.htm.

[5] Franssen L., Coppens J.E., van den Berg T.J.T.P. (2006). Compensation comparison method for assessment of retinal straylight. Investig. Ophthalmol. Vis. Sci..

[6] Coppens J.E., Franssen K., van Rijn L.J., van den Berg T.J.T.P. (2006). Reliability of the compensation comparison straylight measurement method. J. Biomed. Opt..

[7] Coppens J.E., Franssen L., van den Berg T.J.T.P. (2006). Reliability of the compensation comparison method for measuring retinal straylight using Monte-Carlo simulations. J. Biomed. Opt..

[8] Franssen L., Coppens J.E. (2005). Straylight at the Retina—Scattered Papers. Ph.D. Thesis.

[9] Van den Berg T.J.T.P., Spekreijse H. (1999). Light scattering model for donor lenses as a function of depth. Vis. Res..

[10] Franssen L., Tabernero J., Coppens J.E., van den Berg T.J.T.P. (2007). Pupil size and retinal straylight in the normal eye. Investig. Ophthalmol. Vis. Sci..

[11] García-Lázaro S., Ferrer-Blasco T., Ortí-Navarro S., Cerviño A., Montes-Micó R. (2010). Relevance of pupil size in the clinical determination of retinal straylight on young healthy human eyes. Graefes Arch. Clin. Exp. Ophthalmol..

[12] Rozema J.J., van den Berg T.J.T.P., Tassignon M.J. (2010). Retinal Straylight as a Function of Age and Ocular Biometry in Healthy Eyes. Investig. Ophthalmol. Vis. Sci..

[13] Van den Berg T.J.T.P., van Rijn L.R., Michael R., Heine C., Coeckelbergh T., Nischler C., Coppens J.E. (2007). Straylight effects with age and lens extraction. Am. J. Ophthalmol..

[14] Van den Berg T.J.T.P., Franssen L., Kruijt B., Coppens J.E. (2013). History of ocular straylight measurement: A review. Zeitschrift Für Medizinische Physik.

