# Erratum: Longhi Bitencourt, E. et al. South American Values of the Optical Straylight Function. *Vision* 2020, *4*, 2

**DOI:** 10.3390/vision4030042

**Published:** 2020-09-16

**Authors:** Emilia Longhi Bitencourt, Dora Fix Ventura, Marcelo Fernandes Costa

**Affiliations:** 1 Departamento de Psicologia Experimental, Instituto de Psicologia, Universidade de São Paulo, São Paulo 05508030, Brazil; longhi@gmail.com (E.L.B.); dventura@usp.br (D.F.V.); 2 Núcleo de Neurociências e Comportamento e Neurociências Aplicada, Universidade de São Paulo, São Paulo 05508030, Brazil

The authors wish to make the following erratum to this paper [1].

**Request to exclude author Nestor Norio Oiwa from the authorship**. The corrected list of authors should be: Emilia Longhi Bitencourt, Dora Fix Ventura and Marcelo Fernandes Costa.

The change of authorship was caused by a lack of communication between the listed authors and Nestor Norio Oiwa after some changes within the team.

Due to the authorship changes, the authors would like also to request Author Contribution section correction. A corrected Author Contribution section is provided below:

**Author Contributions:** Conceptualization, E.L.B. and M.F.C.; methodology, D.F.V. and M.F.C.; investigation, E.L.B.; resources, D.F.V. and M.F.C.; data curation, E.L.B. and M.F.C.; writing—original draft preparation, E.L.B. and M.F.C.; writing—review and editing, M.F.C.; funding acquisition, D.F.V. and M.F.C. All authors have read and agreed to the published version of the manuscript.

These changes do not influence the results, discussion, or conclusions. The authors would like to apologize for any inconvenience caused to the readers by this error.
